# Supplementary figures and images for: The clinical value of hsa-miR-190b-5p in peripheral blood of pediatric β-thalassemia and its regulation on BCL11A expression
Source: PLoS One. 2023 Oct 5;18(10):e0292031. doi: 10.1371/journal.pone.0292031 (PMC10553837; doi:10.1371/journal.pone.0292031)

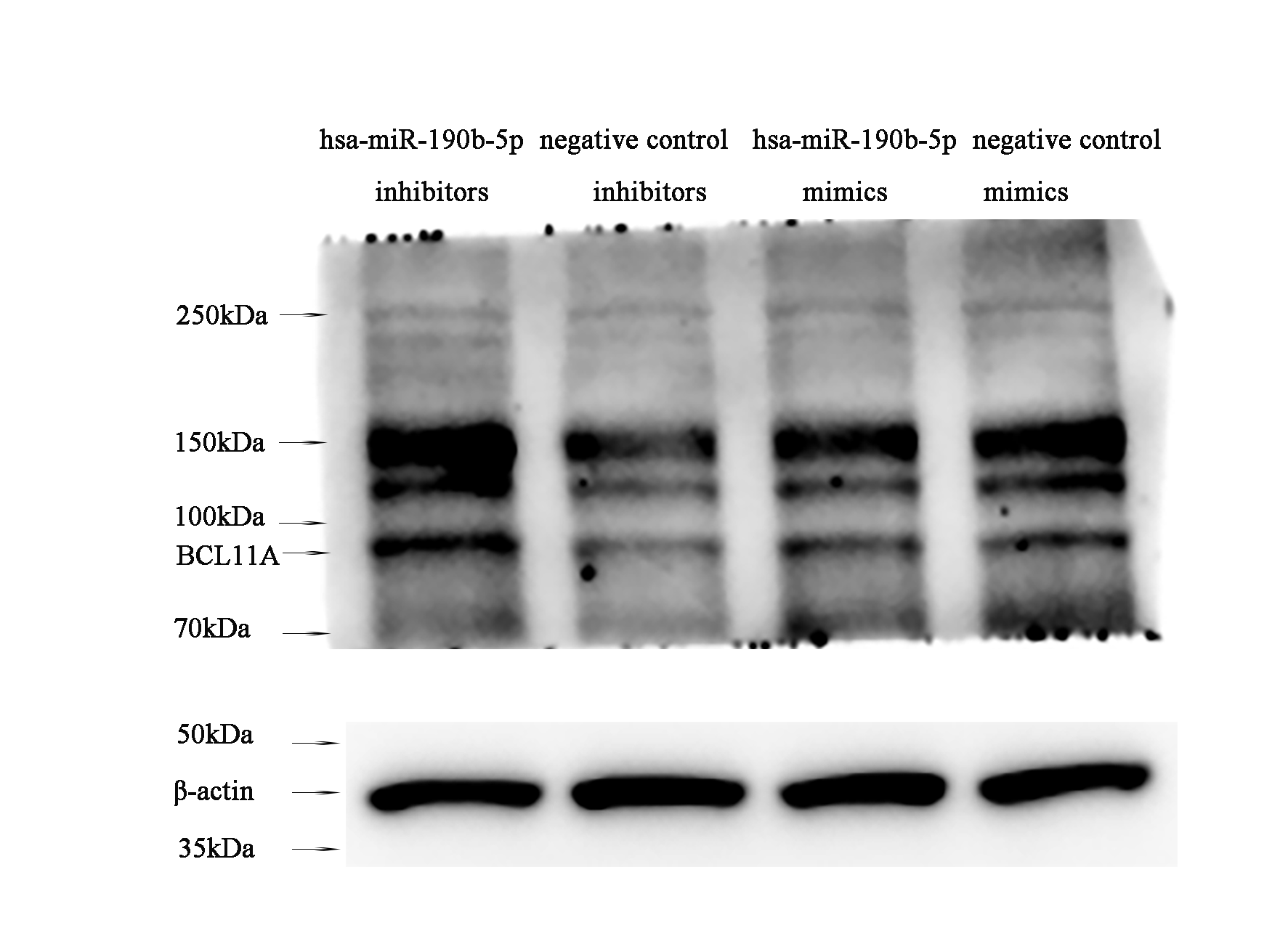

Supplement: S1 Fig — (TIF) [file pone.0292031.s001.tif]

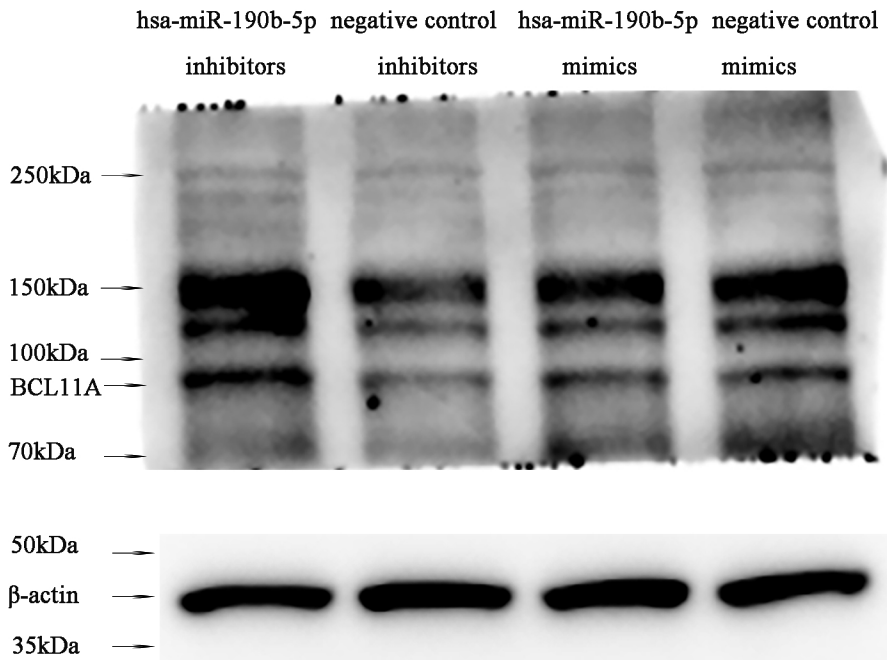

Supplement: S1 Raw images — (PDF) [file pone.0292031.s002.pdf]
