# Supplementary material for: The clinical value of hsa-miR-190b-5p in peripheral blood of pediatric β-thalassemia and its regulation on BCL11A expression
Source: PLoS One. 2023 Oct 5;18(10):e0292031. doi: 10.1371/journal.pone.0292031 (PMC10553837; doi:10.1371/journal.pone.0292031)
Supplement: Attachment — Submitted filename: Response to Reviewers.docx [file pone.0292031.s003.docx]

**Response letter**

[Jun 29 2023]

Dear Editor and reviewers,

First of all, thank you very much for giving us an opportunity to revise our manuscript. We appreciate editor and reviewers for the comments concerning our manuscript entitled “The clinical value of hsa-miR-190b-5p in peripheral blood of pediatric β-thalassemia and its regulation on BCL11A expression” (ID: PONE-D-23-10873). Those comments are all valuable and very helpful for revising and improving our paper. We have studied comments carefully and have made correction which we hope meet with approval. We have thoroughly revised the manuscript accordingly, and changes corresponding to the issues were highlighted in Red in the revised version.

We very much appreciated the valuable comments and suggestions made by the reviewers. We hope that the changes have adequately addressed their concerns. Thank you very much for the time and effort you have spent to help us improve this manuscript.

Sincerely yours,

Hailong Huang

Medical Genetic Diagnosis and Therapy Center of Fujian Maternity and Child Health Hospital College of Clinical Medicine for Obstetrics & Gynecology and Pediatrics, Fujian Medical University, Fujian Provincial Key Laboratory of Prenatal Diagnosis and Birth Defect, Fuzhou 350001, Fujian Province, China

Email: huanghailong@fjmu.edu.cn

**Responses to the Editor**

When submitting your revision, we need you to address these additional requirements.

1. Please ensure that your manuscript meets PLOS ONE's style requirements, including those for file naming. The PLOS ONE style templates can be found at

<https://journals.plos.org/plosone/s/file?id=wjVg/PLOSOne_formatting_sample_main_body.pdf> and <https://journals.plos.org/plosone/s/file?id=ba62/PLOSOne_formatting_sample_title_authors_affiliations.pdf>

**Response:** We ensure that our manuscript meets PLOS ONE's style requirements, including those for file naming.

2. Your ethics statement should only appear in the Methods section of your manuscript. If your ethics statement is written in any section besides the Methods, please delete it from any other section.

**Response:** We ensure that the ethics statement only appears in the Methods section of our manuscript.

3. PLOS ONE now requires that authors provide the original uncropped and unadjusted images underlying all blot or gel results reported in a submission’s figures or Supporting Information files. This policy and the journal’s other requirements for blot/gel reporting and figure preparation are described in detail at <https://journals.plos.org/plosone/s/figures#loc-blot-and-gel-reporting-requirements> and <https://journals.plos.org/plosone/s/figures#loc-preparing-figures-from-image-files>. When you submit your revised manuscript, please ensure that your figures adhere fully to these guidelines and provide the original underlying images for all blot or gel data reported in your submission. See the following link for instructions on providing the original image data: <https://journals.plos.org/plosone/s/figures#loc-original-images-for-blots-and-gels>.  In your cover letter, please note whether your blot/gel image data are in Supporting Information or posted at a public data repository, provide the repository URL if relevant, and provide specific details as to which raw blot/gel images, if any, are not available. Email us at [plosone@plos.org](mailto:plosone@plos.org) if you have any questions

**Response:** We ensure that our original uncropped and unadjusted images underlying blot/gel image data are shown in Supporting Information.

4. PLOS requires an ORCID iD for the corresponding author in Editorial Manager on papers submitted after December 6th, 2016. Please ensure that you have an ORCID iD and that it is validated in Editorial Manager. To do this, go to ‘Update my Information’ (in the upper left-hand corner of the main menu), and click on the Fetch/Validate link next to the ORCID field. This will take you to the ORCID site and allow you to create a new iD or authenticate a pre-existing iD in Editorial Manager. Please see the following video for instructions on linking an ORCID iD to your Ed **Response:** We ensure that the corresponding author ORCID Id (0000-0001-5775-5082) is validated in Editorial Manager account.

Additional Editor Comments :
5.Authors should list the limitations of the study properly in the discussion. When listing the limitations of a study, authors should be specific and provide details about how the limitations might have affected the findings. They should also discuss how the limitations could be addressed in future research.
**Response:** We are very appreciated for this comment. We have listed the limitations of the study in the discussion in Page 16, line 10-17 as “The shortcomings of the current study were as follows: 1) The sample size enrolled in this study was small, which might have limited generalizability. Large-scale clinical trials were needed to further validate the expression level and clinical value of hsa-miR-190b-5p in pediatric β-thal. 2) The role of hsa-miR-190b-5p in the differentiation of erythroid precursor cells was unknown, which would affect the determination of whether it could be provided as an effective strategy for pediatric β-thal treatment. Further studies were necessary to fucus on its role and relationship with BCL11A in erythroid precursor cells and in extensive animal experiments.”

**Responses to Reviewer #1’s comments**

Reviewer #1: The manuscript has been thoroughly reviewed, and no further comments or revisions have been proposed, indicating a recommendation for acceptance. However, it is important to address two specific issues raised during the review process.:
Firstly, the study's small sample size should be acknowledged as a potential limitation and discussed in the appropriate section.
**Response:** We are very appreciated for this comment. We have listed the “the study's small sample size” as a limitation of the study in the discussion in Page 16, line 10-13 as “The shortcomings of the current study were as follows: 1) The sample size enrolled in this study was small, which might have limited generalizability. Large-scale clinical trials were needed to further validate the expression level and clinical value of hsa-miR-190b-5p in pediatric β-thal.”

Additionally, the quality of the figures needs improvement, and it is advised to replace them with higher-quality images to enhance visual clarity and data presentation
**Response:** We are very appreciated for this comment. We have replaced these figures with higher-quality to enhance visual clarity and data presentation.

**Responses to Reviewer #2’s comments**

Reviewer #2: This manuscript satisfies the PLOS ONE criteria for publication as it is an original manuscript with added knowledge in the field. The results presented have not been published elsewhere. The authors well described the methodology and results with sufficient details. This study has received ethical clearance from the Ethics Review Committee of Fujian Province Maternity and Child Health Hospital (approval no. 073, 2019), and this study has been conducted with adherence to the Declaration of Helsinki. This manuscript has provided new information on β-thal disease by highlighting the role of the hsa-miR-19b-5p in ameliorating the β-thal through its direct action on BCL11A to downregulate BCL11A and thus increase the HbF level in β-thal patients. The potential of hsa-miR-190b-5p as a biomarker for β-thal was evaluated by the authors, who suggested that hsa-miR-190b-5p is similarly important as HbA2 for discriminating pediatric β-thal patients from healthy controls. The authors also proved the capability of hsa-miR-19b-5p direct binding to BCL11A. The authors have clearly and thoroughly discussed their findings with relevant references. Some grammatical errors may be corrected prior to publication.

1) Page 8, recheck this sentence: "Compared with healthy controls, the levels of red blood cells (RBC) and hemoglobin (Hb) in pediatric β-thal patients (p<0.001).";

**Response:** We are very appreciated for this comment. We have rechecked this sentence “Compared with healthy controls, the levels of red blood cells (RBC) and hemoglobin (Hb) in pediatric β-thal patients (p<0.001).” and change it to “Compared with healthy controls, the levels of red blood cells (RBC) and hemoglobin (Hb) were significantly lower in pediatric β-thal patients (*p*<0.001)” in Page 8, line 16.
2) Page 12, recheck this sentence: "The above data hinted that that decreased hsa-miR-190b-5p has potential as a diagnostic biomarker for pediatric β-thal.";

**Response:** We are very appreciated for this comment. We have rechecked this sentence “The above data hinted that that decreased hsa-miR-190b-5p has potential as a diagnostic biomarker for pediatric β-thal.” and change it to “The above data hinted that decreased hsa-miR-190b-5p could be used as a potential diagnostic biomarker for pediatric β-thal.” in Page 15, line 13.

3) Figure 1, recheck this sentence: "Regulation of hsa-miR-190b-5p on the garget gene BCL11A.".

**Response:** We are very appreciated for this comment. We have rechecked this sentence “Regulation of hsa-miR-190b-5p on the garget gene BCL11A.” and change it to “Regulation of hsa-miR-190b-5p on the target gene BCL11A.” in revised Figure 1.
