# Supplementary material for: The clinical value of hsa-miR-190b-5p in peripheral blood of pediatric β-thalassemia and its regulation on BCL11A expression
Source: PLoS One. 2023 Oct 5;18(10):e0292031. doi: 10.1371/journal.pone.0292031 (PMC10553837; doi:10.1371/journal.pone.0292031)
Supplement: Attachment — Submitted filename: Response to Reviewers.docx [file pone.0292031.s004.docx]

**Response letter**

[Sept 1 2023]

Dear Editor and reviewers,

First of all, thank you very much for giving us an opportunity to revise our manuscript. We appreciate editor and reviewers for the comments concerning our manuscript entitled “The clinical value of hsa-miR-190b-5p in peripheral blood of pediatric β-thalassemia and its regulation on BCL11A expression” (ID: PONE-D-23-10873R1). Those comments are all valuable and very helpful for revising and improving our paper. We have studied comments carefully and have made correction which we hope meet with approval. We have thoroughly revised the manuscript accordingly, and changes corresponding to the issues were highlighted in Red in the revised version.

We very much appreciated the valuable comments and suggestions made by the reviewers. We hope that the changes have adequately addressed their concerns. Thank you very much for the time and effort you have spent to help us improve this manuscript.

Sincerely yours,

Hailong Huang

Medical Genetic Diagnosis and Therapy Center of Fujian Maternity and Child Health Hospital College of Clinical Medicine for Obstetrics & Gynecology and Pediatrics, Fujian Medical University, Fujian Provincial Key Laboratory of Prenatal Diagnosis and Birth Defect, Fuzhou 350001, Fujian Province, China

Email: huanghailong@fjmu.edu.cn

**Journal Requirements:**

Please review your reference list to ensure that it is complete and correct. If you have cited papers that have been retracted, please include the rationale for doing so in the manuscript text, or remove these references and replace them with relevant current references. Any changes to the reference list should be mentioned in the rebuttal letter that accompanies your revised manuscript. If you need to cite a retracted article, indicate the article’s retracted status in the References list and also include a citation and full reference for the retraction notice.

**Response:** All references cited in our manuscript have not been retracted. All references cited in our manuscript have been formatted according to the requirements of PLOS ONE.

**Responses to Reviewer #3’s comments**

Reviewer #3: Nice paper

would have been interesting to see if miR190 was not influenced by anemia out or thalassemia

**Response:** We are very appreciated for this comment. In the future, we will be collaborating with other research groups to investigate the expression and clinical value of hsa-miR-190b-5p in other hematological diseases, such as leukemia, multiple myeloma, aplastic anemia.

**Minor comments:**

Page 3 line 24 “…In recently, several…” what does this means?

**Response:** The meaning of this sentence is that BCL11A is key regulator for HbF expression, and other transcription factor (KLF1, KLF1 and MYB) could impact HbF expression via regulation of BCL11A. This sentence is a bit vague, we've corrected it.

Page 13 line 3“…was negative regulated…” do you mean negatively regulated?

**Response:** Thanks for the reminder. It was a grammatical error and we've corrected it.
